# The genetic correlation between feed conversion ratio and growth rate affects the design of a breeding program for more sustainable fish production

**DOI:** 10.1186/s12711-020-0524-0

**Published:** 2020-02-07

**Authors:** Mathieu Besson, Hans Komen, Gus Rose, Marc Vandeputte

**Affiliations:** 1 Animal Breeding and Genomics Centre, Wageningen University, PO Box 338, 6700 AH Wageningen, The Netherlands; 2 Université Paris-Saclay, INRAE, AgroParisTech, GABI, 78350 Jouy-en-Josas, France; 3 Ifremer, Chemin de Maguelone, 34250 Palavas-les-Flots, France

## Abstract

**Background:**

Most fish breeding programs aim at improving growth rate and include feed conversion ratio (FCR) neither in the breeding goal nor in the selection index, although decreasing FCR is known to increase farm profit and decrease environmental impacts. This is because FCR is difficult to measure in fish that live in groups and FCR is assumed to have a favourable (negative) genetic correlation with growth, although the magnitude of this correlation is unknown. We investigated the effect of the genetic correlation between growth and FCR on the economic and environmental responses of a two-trait breeding goal (growth and FCR), compared to a single-trait breeding goal (growth only). Next, we evaluated the weights to assign to growth and FCR in a two-trait breeding goal to maximize sustainability of fish production.

**Methods:**

We used pseudo-best linear unbiased prediction (BLUP) index calculations to simulate a breeding program for sea bass. For the single-trait breeding goal, the trait in the breeding goal and in the index was thermal growth coefficient (TGC) and for the two-trait breeding goal, the traits in the breeding goal were TGC and FCR and the traits in the index were TGC and percentage of fat in the dorsal muscle (an indirect measure of FCR). We simulated responses to selection for genetic and phenotypic correlations between TGC and FCR ranging from 0 to − 0.8. Then, in the two-trait breeding goal, we calculated the economic return and the change in eutrophication when using economic values (EV) or environmental values (ENV).

**Results:**

When the genetic correlation between TGC and FCR was lower than − 0.45, we found major differences in economic returns and in eutrophication between single and two-trait breeding programs. At a correlation of − 0.25, the two-trait breeding goal based on EV increased economic return by 25% compared to the single-trait breeding goal, while using ENV decreased eutrophication by 1.34% per ton of fish produced after one generation of selection.

**Conclusions:**

The genetic correlation between TGC and FCR affects the magnitude of economic losses due to omitting FCR in the breeding program. In addition, the genetic correlation affects the importance of choosing EV or ENV to reduce eutrophication and increase profit.

## Background

Most fish breeding companies consider growth rate as the major trait to be improved in their breeding program [1]. When a farm is operating under a quota on biomass, which is for example the case for salmon farms in Norway, improving growth rate is expected to increase farm profit through a reduction in production time, thus increasing annual production and returns. However, livestock and fish production has an impact on the environment [2, 3], which raises the need for breeding programs that reduce these impacts. Several studies have already investigated the environmental impact of genetic improvement of traits in livestock [4–7] and fish production [8, 9]. Our studies on fish showed that improving feed conversion ratio (FCR; the ratio of feed intake over body weight gain) can increase profit and decrease environmental impacts at the same time, which makes ${\text{FCR}}$ an essential trait to include in breeding programs. However, unlike terrestrial livestock, feed efficiency is typically not included in fish breeding programs because individual feed intake cannot be measured accurately in group-reared fish (see review in [10]), and because feed efficiency is assumed to have a favourable (negative) correlation with growth rate, although the exact value of this correlation is uncertain. Several studies in terrestrial animals and in fish have reported a negative genetic correlation between growth and ${\text{FCR}}$ [11, 12], whereas other studies on fish showed a zero correlation (e.g. in brown trout [13–15]).

The genetic response of a breeding program depends on the phenotypic and genetic correlations between the traits in the breeding goal and in the corresponding selection index. In the case of a single-trait breeding goal where the trait of interest is e.g. growth rate, the response depends only on the heritability and phenotypic variance of growth rate, and on the intensity of selection. A correlated response in FCR will depend on the genetic standard deviation of ${\text{FCR}}$ and on the genetic correlation between ${\text{FCR}}$ and growth rate. In a breeding goal with growth rate and FCR, the response depends not only on the phenotypic and genetic correlations between the traits in the selection index and in the breeding goal but also on the weights applied to the traits in the breeding goal (Eq. 3, see below). When the main objective of selection is to maximise farm profit, the weights used in the breeding goal are economic values (EV). Using these values in a breeding goal and the optimal index corresponding to that breeding goal optimizes the direction and magnitude of the genetic responses in growth rate and FCR to maximize the economic return of genetic improvement.

However, EV might not be the best weights to enhance the environmental sustainability of fish production. In a previous study [8], we calculated environmental values (ENV) of fish traits by combining bio-economic modelling and life cycle assessment (LCA) [16], as in van Middelaar et al. [6]. Similar to EV, ENV express the change in different categories of environmental impact (e.g. climate change or eutrophication) when changing one trait and keeping the other traits in the breeding goal constant. These ENV can be used as weights in the breeding goal to derive a selection index that maximizes the reduction of the environmental impacts of fish production. In this study, we calculated EV based on the impact of genetic change on profit per farm per year and ENV based on differences in kg of pollutants emitted per farm per year (Eqs. (1) and (2), see below). We chose these units because most pricing mechanisms, constraints on inputs and outputs, and management variables act at the farm level [17]. At the farm level, the ENV consider the absolute change in environmental impacts to reflect the environmental impact of a farming site (i.e. benthos degradation, dissolved nutrient emissions, ecosystem changes).

In this paper, we explored different strategies to enhance the economic and environmental sustainability of fish production. First, we explored the potential gain in economic return of upgrading a simple breeding program for growth rate only by including FCR in the breeding goals and percentage of fat in the dorsal muscle as an indirect criterion of FCR in the index. We explored this potential gain as a function of the genetic and phenotypic correlation between growth and ${\text{FCR}}$. Then, we compared the response to selection in terms of economic gains and change in eutrophication for the two-trait (growth and ${\text{FCR}}$) breeding goal using economic (EV) or environmental weights (ENV).

## Methods

In a previous study [9], we calculated EV and ENV for thermal growth coefficient $\left( {{\text{TGC}}\;{\text{in}}\;{\text{g}}^{1/3} \cdot {\text{d}}^{ - 1} \cdot {\text{C}}^{ - 1} } \right)$ and ${\text{FCR}}$ using a bio-economic model and an LCA for sea bass reared in sea cages. The approach and models used are briefly described below.

### Bio-economic model

The bio-economic model estimated the production of sea bass (*Dicentrarchus labrax*) in a hypothetical sea cage farm producing 1000 tons of sea bass per year, where the instant biomass present on site was constrained to 435 tons (“standing stock” or “biomass” quota). The farm was composed of 34 circular cages of 600 m^3^ for pre-growing and 34 circular cages of 1800 m^3^ for on-growing. Fish were stocked at 10 g and sold at a fixed harvest weight of 400 g. Stocking occurred all year round. The bio-economic model was divided into four model parts.

1. The fish model estimates individual fish growth using ${\text{TGC}}$ corrected for the concave relationship between growth rate and temperature [18]. ${\text{FCR}}$ was modelled by combining a third order polynomial model from Person-Le Ruyet et al. [19] that models FCR as a function of temperature at a fixed body weight with an exponential model from Lanari et al. [20] that models the variation of FCR with fish body weight. The fish model also estimates the individual emission of nutrient-based pollutants using mass-balance [21, 22].
2. The batch model estimates the average stocking density of a batch depending on individual fish performances (from the fish model) and mortality. A batch is defined as the group of fish stocked at the same time in the same pre-growing cage.
3. The farm model estimates the number of batches produced to calculate annual fish production, emission of pollutants, and annual feed consumption, while complying with the quota on biomass.
4. Finally, in the economic model, annual profit is calculated by combining results of the farm model with economic parameters.

Further details about the bio-economic model are in Additional files 1, 2, 3: Tables S1, S2 and S3.

### Life cycle assessment

LCA is a standardized method to calculate the environmental impact of a production chain, from raw material extraction up to the product’s end of life [23]. The production chain studied here included five distinct sub-systems: (1) production of purchased feed, including production of ingredients, processing, and transportation; (2) production of energy expended at the farm level (electricity, gas and petrol); (3) production of farming facilities and equipment; (4) chemicals used, including the production and use of anti-fouling for nets; (5) farming operations, including emission of nutrient based pollutants from biological transformation of feed.

Each flow of resources and pollutants observed in the system was assigned to eutrophication potential. We chose to investigate only eutrophication because quotas are essentially designed to limit the eutrophication caused by fish farming. The characterization factors in the CML2 Baseline 2000 version 2.04 method were used to compute eutrophication. The categories of impact were calculated using the Simapro^®^ 7.0 software. Eutrophication was expressed per farm on the basis of 1 year of routine production (impact_farm). The impact_farm values were subsequently used to calculate ENV.

### Economic and environmental values

The EV and ENV of a trait were calculated for a one genetic standard deviation change in the mean of the trait while the means of the other traits remained constant. When calculating EV and ENV, increasing ${\text{TGC}}$ while keeping ${\text{FCR}}$ constant was achieved by increasing feed intake. Conversely, improving ${\text{FCR}}$ while keeping ${\text{TGC}}$ constant was generated by reducing feed intake.

Unlike previous studies [9, 24], we displayed EV as monetary gain per one unit of trait change (and not per genetic standard deviation), i.e. from 2.25 to 3.25 ${\text{g}}^{1/3} \cdot {\text{d}}^{ - 1} \cdot {\text{C}}^{ - 1}$ for ${\text{TGC}}$ and from 2.03 to 1.03 for ${\text{FCR}}$, to comply with the requirements of the software (SelAction) used to compute response to selection. The EV of a trait was calculated as the difference between profit before (${\text{profit}}\_{\text{before}}$) and after (${\text{profit}}\_{\text{after}}$) changing the trait by one unit, divided by the production of fish before genetic change (${\text{production}}\_{\text{before}}$).

1 $${\text{EV }} = \frac{profit\_after - profit\_before}{production\_before}.$$

We used the eutrophication per year per farm ($impact\_farm$) to calculate environmental values for eutrophication at the farm level for ${\text{TGC}}$ and ${\text{FCR}}$. The ENV of a trait was calculated as the difference between $impact\_farm$ before ($impact\_farm\_before)$ and after genetic change ($impact\_farm\_after$) changing the trait by one trait unit, divided by the production of fish before genetic change. Thus, the ENV refers to the local environmental impacts caused by a farm.

2 $${\text{ENV}} = \frac{impact\_farm\_after - impact\_farm\_before}{production\_before}.$$

The resulting EV and ENV are in Table 1. Here, we consider that a positive EV or ENV means that an increase in trait value increases economic return and decreases environmental impacts of a farm.

**Table 1:** Economic (EV) and environmental values at the farm level (ENV) of thermal growth coefficient $\left( {\text{TGC}} \right)$ and feed conversion ratio ($\text{FCR}$) expressed per unit of change in each trait

	$\text{TGC}$	$\text{FCR}$	Ratio (EV_TGC_/EV_FCR_)
EV (€/kg of fish produced)	0.65	− 1.32	1: − 2.03
ENV (g PO_4_-eq/kg fish produced)	− 48.83	− 106.67	1: 2.18

The mechanisms by which a change in TGC determined its EV and ENV were as follows. An increase in TGC reduces the production cycle and therefore, increases the number of times per year when the farm is running at the maximum biomass [9]. Therefore, improving TGC increases production and increases the number of juveniles purchased. Furthermore, at a constant FCR, an increase in TGC does not affect total feed intake over the life of a fish but annual feed consumption per year per farm increases due to higher production. Consequently, the EV of TGC is positive because extra profit from higher production overtakes extra costs of feed and juveniles. However, the ENV of TGC is negative because an increase in TGC increases eutrophication due to greater use of feed and greater emissions of pollutants per farm per year [9]. The mechanism by which changes in FCR determined its EV and ENV was that a reduction of FCR while keeping TGC constant reduces the total amount of feed required to reach harvest weight. Therefore, reducing FCR reduces the annual use of feed per farm [9]. Consequently, the EV and ENV of FCR are both negative, meaning that an increase in FCR decreases profit and eutrophication.

### Simulated breeding program

We simulated a simple breeding program for sea bass using SelAction [25], in which 100 females were mated to 100 males to create 100 full-sib families. Forty fish (20 females and 20 males) were kept per family (4000 fish in total) as selection candidates. From these candidates, 200 (5%, 100 males and 100 females) were selected as parents for the next generation, corresponding to a selection intensity of 2.06. The breeding goal included two traits, ${\text{TGC}}$ and ${\text{FCR}}$:

3 $${\text{H}} = {\text{W}}_{\text{TGC}} \times {\text{A}}_{\text{TGC}} + {\text{W}}_{\text{FCR}} \times {\text{A}}_{\text{FCR}} ,$$

where, ${\text{W}}$ is the EV or ENV and ${\text{A}}$ is the additive genetic value. Selection was on a pseudo-BLUP selection index based on own performance and information from 39 full sibs for ${\text{TGC}}$ and the percentage of fat in dorsal muscle (%fat). We assumed a non-lethal measurement of  %fat using ultrasounds as an indirect criterion of ${\text{FCR}}$ as in Kause et al. [26]. Genetic gain per generation obtained from SelAction was converted to genetic standard deviations (σ_g_) per year considering an average generation interval of 2.5 years (3 years for females and 2 years for males). We expressed genetic gain in σ_g_ to compare the genetic gain achieved for the three traits on a standardized basis.

We used the single trait breeding goal ${\text{H}} = {\text{A}}_{\text{TGC}}$ as the baseline where selection was on ${\text{TGC}}$ using a pseudo-BLUP index based on own performance and information from 39 full sibs for ${\text{TGC}}$ only, resulting in correlated responses in %fat and ${\text{FCR}}$.

### Genetic parameters

Genetic parameters of the three traits are in Tables 2 and 3. For FCR, genetic parameters were from rainbow trout (*Oncorhynchus mykiss*), whereas correlations between ${\text{FCR}}$ and % fat were from European sea bass. Genetic and phenotypic correlations between ${\text{TGC}}$ and ${\text{FCR}}$ are uncertain in sea bass. Thus, we tested values ranging from 0 to − 0.8 in steps of 0.01 for both genetic and phenotypic correlations between ${\text{TGC}}$ and ${\text{FCR}}$. The genetic and phenotypic correlations between TGC and FCR were assumed equal to each other.

**Table 2:** Genetic parameters of thermal growth coefficient (${\text{TGC}}$), feed conversion ratio ($\text{FCR}$) and percentage of muscle fat (% fat) used to simulate response to selection

Trait	Heritability	Genetic standard deviation	References
TGC	0.43	0.23	[27]
${\text{FCR}}$	0.17	0.38	[12]
% fat	0.42	1.18	[26]

**Table 3:** Genetic (above diagonal) and phenotypic (below diagonal) correlations between thermal growth coefficient (${\text{TGC}}$), feed conversion ratio ($\text{FCR}$) and percentage of muscle fat (%fat)

	${\text{TGC}}$	${\text{FCR}}$	%fat
${\text{TGC}}$		[− 0.8:0]^a^	0.75^b^
${\text{FCR}}$	[− 0.8:0]^a^		− 0.39^b^
%fat	0.31^b^	− 0.02^b^	

^a^Correlations values between brackets refer to the range of values tested from 0 to − 0.8 with a step of 0.01

^b^Based on [33]

## Results

### Genetic gain

**Fig. 1:**
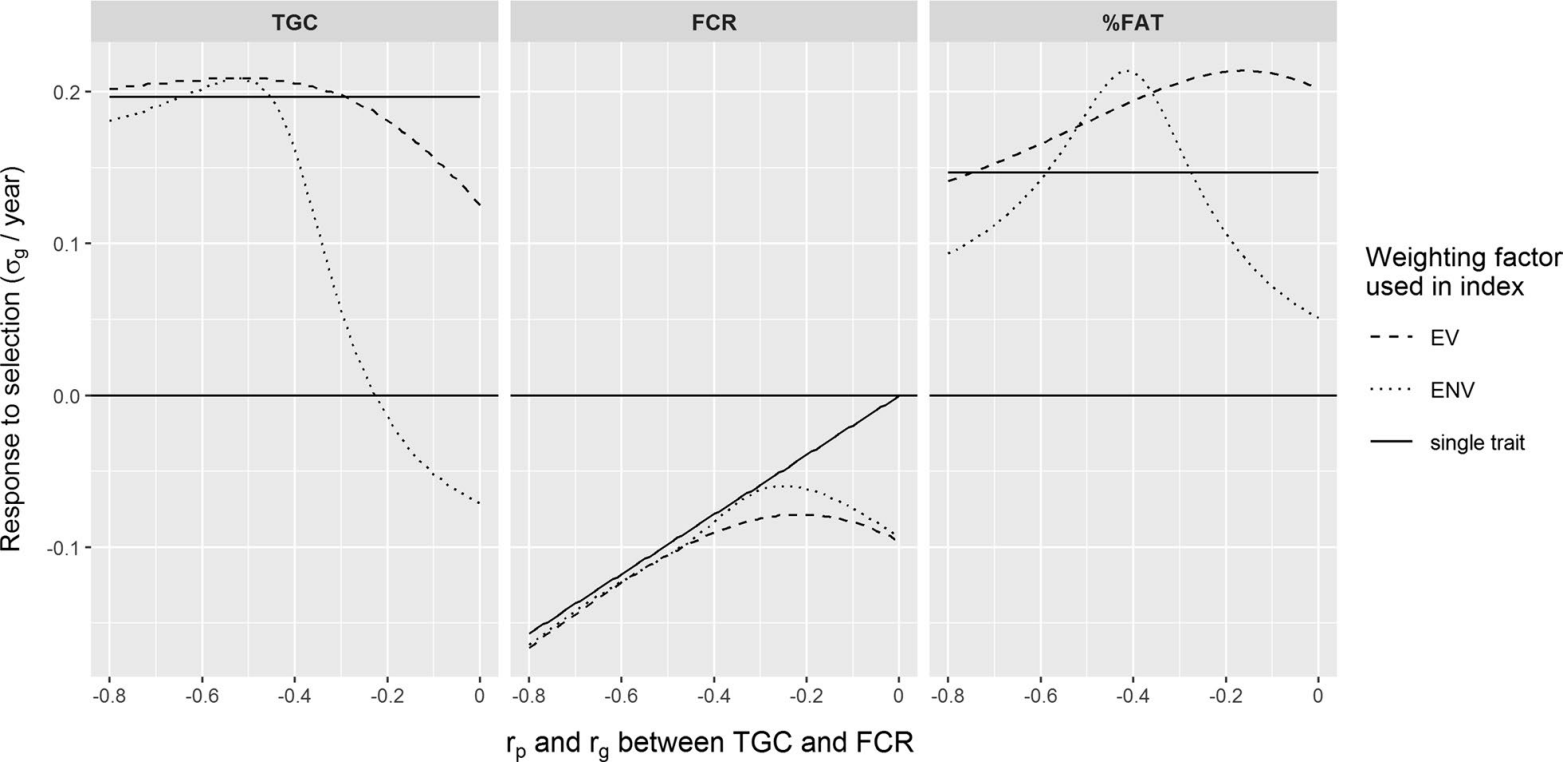
Response to selection for thermal growth coefficient ($\text{TGC}$) (left panel), feed conversion ratio ($\text{FCR})$ (middle panel) and %fat (right panel) as a function of the genetic correlation (r_g_) between $\text{TGC}$ and $\text{FCR}$, for a single-trait breeding goal with $\text{TGC}$ (“single trait”) and for breeding goals with $\text{TGC}$ and $\text{FCR}$ weighted by EV and ENV, respectively. Values are expressed in genetic standard deviations (σ_g_) per year

In the single-trait breeding goal, the response to selection for ${\text{TGC}}$ was always the same regardless of the correlation with ${\text{FCR}}$ because only the response for ${\text{TGC}}$ was maximized (Fig. 1, left panel). The correlated response for %fat was also constant since the genetic correlation between TGC and %fat was fixed (Fig. 1, right panel). Conversely, as expected, the correlated response in FCR from selection on ${\text{TGC}}$ was higher when the genetic correlation between TGC and FCR was stronger (Fig. 1, central panel).

In a two-trait breeding goal, the response to selection achieved is the result of a complex interaction between weights assigned to each trait and the additive genetic variances for those traits, and their correlations. The response to selection for ${\text{FCR}}$ was favourable (FCR decreased) and similar when using either EV or ENV (Fig. 1, central panel). When the correlation between ${\text{TGC}}$ and ${\text{FCR}}$ was strongly negative (< − 0.17 for EV and < − 0.22 for ENV), response to selection for ${\text{FCR}}$ increased because FCR could be improved by simply improving TGC. When the correlation between ${\text{TGC}}$ and ${\text{FCR}}$ reached − 0.8, almost all the selection response for ${\text{FCR}}$ was due to the improvement in ${\text{TGC}}$ and there was little benefit from including %fat. However, the improvement in ${\text{FCR}}$ reached a minimum value when the correlation between ${\text{TGC}}$ and ${\text{FCR}}$ was − 0.17 (when using EV) or − 0.22 (when using ENV).

Interestingly, using EV or ENV caused different responses for ${\text{TGC}}$ (Fig. 1, left panel). For correlations of ${\text{TGC}}$ with ${\text{FCR}}$ between − 0.21 and 0, TGC decreased when using ENV basically because ${\text{TGC}}$ was quite heritable (h^2^ = 0.43) and because the ENV of ${\text{TGC}}$ was negative. When the correlations became stronger (< − 0.21), improving ${\text{FCR}}$, which was the trait with the largest ENV could only be achieved by increasing TGC. On the contrary, the response to selection for ${\text{TGC}}$ was always positive (TGC increased) when using EV because the EV of ${\text{TGC}}$ was positive and selection on ${\text{TGC}}$ generated a favourable correlated response for ${\text{FCR}}$ (except when the correlation was exactly 0).

As expected, response for %fat was positive when using EV due to the positive correlation of ${\text{TGC}}$ with %fat (Fig. 1, right panel). The increase in %fat was even larger when the correlations between ${\text{TGC}}$ and ${\text{FCR}}$ approached 0, due to the increased importance of %fat to improve ${\text{FCR}}$. When using ENV, the increase in response for %fat was largest for correlations between ${\text{TGC}}$ and ${\text{FCR}}$ of − 0.4. For correlations greater than − 0.4, the response in  %fat decreased in order to generate a decrease in ${\text{TGC}}$, which had a negative ENV. For correlations lower than − 0.4, the response for %fat decreased because the correlations between ${\text{TGC}}$ and ${\text{FCR}}$ were sufficiently high to generate a favourable correlated response for ${\text{FCR}}$ without having to increase %fat too much.

### Economic return and change in eutrophication

**Fig. 2:**
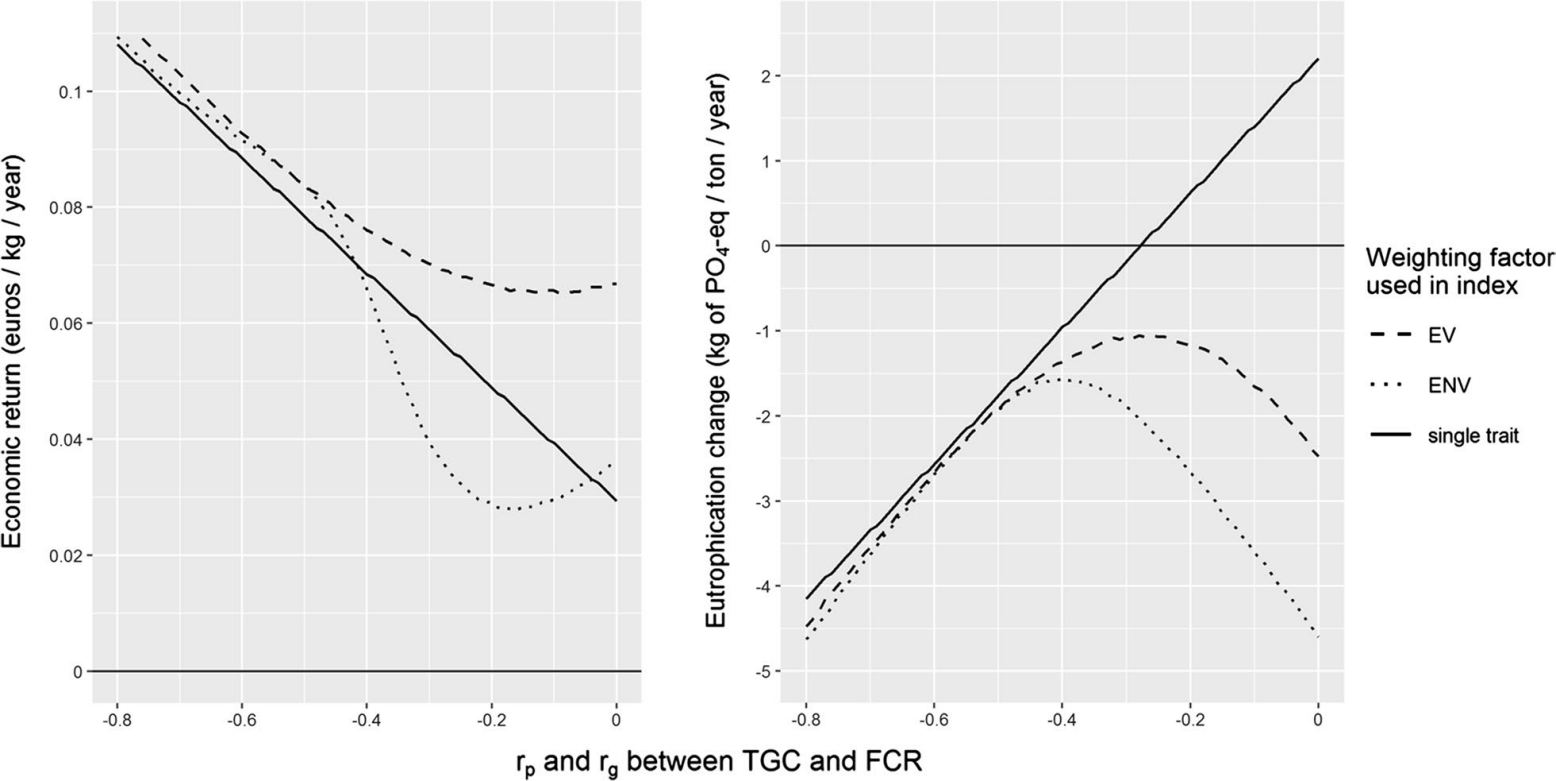
Economic (left panel) and environmental (right panel) response to selection as a function of genetic correlation (r_g_) between thermal growth coefficient ($\text{TGC}$), feed conversion ratio ($\text{FCR}$). The economic and environmental responses were calculated for a single-trait breeding goal with ${\text{TGC}}$ (“single trait”) and for breeding goals with ${\text{TGC}}$ and ${\text{FCR}}$ weighted by EV or ENV. Values are expressed as economic return (euros per kg of fish produced per year) or reduction in eutrophication (kg PO_4_-eq per ton of fish produced per year)

With the single-trait breeding goal, economic returns increased linearly (Fig. 2, left panel) while eutrophication decreased linearly (Fig. 2, right panel) with a decrease in the correlation between ${\text{TGC}}$ and ${\text{FCR}}$. Nevertheless, implementing a single-trait breeding goal caused an increase in eutrophication per farm per year when the correlation between ${\text{TGC}}$ and ${\text{FCR}}$ was weak, between − 0.27 and 0 (Fig. 2, right panel).

In contrast, for the two-trait breeding goal, using either EV or ENV increased economic return and decreased eutrophication. As expected, using EV in the breeding goal gave the greatest economic return (Fig. 2, left panel). Economic returns were similar between EV and ENV when the correlation between ${\text{TGC}}$ and ${\text{FCR}}$ was lower than − 0.5. However, when the correlation was between − 0.5 and 0, the economic return achieved when using ENV was lower than when using EV. The difference in economic return between EV and ENV reached a maximum when the correlation between ${\text{TGC}}$ and ${\text{FCR}}$ was − 0.19 (0.038 €/kg produced/year). Note that, when the correlation was between − 0.41 and − 0.05, using ENV resulted in lower economic returns than the single-trait breeding goal.

Using ENV in the breeding goal generated a reduction of eutrophication of at least 1.55 kg PO_4_-eq per ton of fish produced per year (Fig. 2, right panel, correlation − 0.4). This is a reduction of 0.92% per year, considering an eutrophication of 168.51 kg PO_4_-eq per kg produced per year before genetic improvement [9]. With correlations closer to zero, the reduction of eutrophication rapidly reached 4.5 kg PO_4_-eq per ton of fish produced per year, which is more than what was obtained by using EV (2.5 kg PO_4_-eq with a correlation of 0). The reduction in eutrophication per year did not differ between EV and ENV when the correlation between ${\text{TGC}}$ and ${\text{FCR}}$ was lower than − 0.45.

## Discussion

To our knowledge, this is the first study that explores the influence of the correlation between growth rate (expressed as ${\text{TGC}}$) and ${\text{FCR}}$ on the design of a fish breeding program for economic or environmental sustainability. Although selection on a component trait such as ${\text{FCR}}$ is generally assumed to be less efficient than selection on an index weighing the components, selection on ${\text{FCR}}$ directly could be more efficient if the heritabilities of both traits (body weight gain and feed intake) were similar [29]. In fish, data on the genetic parameters of feed intake are still lacking and the best strategy to maximize improvement of feed efficiency is yet to be determined. Measuring ${\text{FCR}}$ directly on individual fish is indeed difficult and improving ${\text{FCR}}$ depends on its correlation with other traits included in the breeding goal and in the index. In fish, the genetic correlation of FCR with ${\text{TGC}}$, the trait considered as most important by farmers, is uncertain. Thus, we explored the effect of the correlation between ${\text{TGC}}$ and ${\text{FCR}}$ on the response to selection and on the economic return of two breeding programs: (1) single-trait breeding goal, the trait in the breeding goal and in the index was TGC; and (2) a two-trait breeding goal, where TGC and FCR were in the breeding goal while TGC and percentage of fat in the dorsal muscle (%fat) were in the index. In this index, %fat was used as an indirect criterion of FCR. Then, for the two-trait breeding goal, we explored the effect of this correlation between TGC and FCR on the economic return and on the eutrophication change when using economic values or environmental values as weights in the breeding goal.

According to Brascamp et al. [30], the economic values of traits should be calculated while considering that the farm is running under an optimized state and that, in the long term, extra profit from increasing production tends to be absorbed by the different stakeholders of an industry. Smith et al. [31] added that, in such industries where an equilibrium is reached, only decreases in cost should be included in the calculation of economic values. In the present study, harvest weight was fixed at 400 g, and the technical (number of cages) and zootechnical parameters (stocking density) were optimized to produce 1000 tons while keeping the constraint on the biomass. However, we decided to include the extra profit due to higher production in the calculation of economic values because fish farming is a recent industry and is not at equilibrium due to constant innovations. For a growing industry such as fish farming, any improvement of production volume within the production system and its quotas should be considered, as it reflects better production efficiency. This extra profit generated by increasing production could then be reinvested to fuel these innovations. This is supported by Amer et al. [32] who suggested that economic values depend on the economic and technical context of the industry.

Our results show that there are only minor differences in economic and environmental responses between the single-trait breeding goal and the two-trait breeding goal based on EV or ENV when the genetic correlation between FCR and TGC is strongly negative (< − 0.5). This suggests that, in such cases, an easy and affordable single-trait breeding program for ${\text{TGC}}$ only should be sufficient to generate economic profit and simultaneously reduce environmental impacts, although it does not maximize the economic or environmental responses. The reason for this small difference between single and two-trait breeding goals is that improving ${\text{TGC}}$ is easy (due to its high heritability), and indirectly generates a favourable correlated response for ${\text{FCR}}$. However, when the correlation between ${\text{TGC}}$ and ${\text{FCR}}$ is weaker (0 to − 0.5), there are large differences in economic return and in reduction of environmental impacts between single and two-trait breeding goals. A breeding program with only ${\text{TGC}}$ in the index performs less well in terms of economic return than a breeding program with TGC and %fat in the index using EV as weights in the breeding goal. This difference is a direct result of the introduction of %fat in the index that allowed to improve the response to selection in FCR in the two-trait breeding goal. For instance, if the correlation is around − 0.2, the economic return is 0.066 €/kg produced/year with the two-trait breeding goal and 0.049 €/kg with the single-trait breeding goal. Thus, this represents a reduction of about 26.6% of the economic return. This reduction is even larger when the correlation is null. This difference between single and two-trait breeding goals is also observed for the reduction of eutrophication. With a correlation of − 0.4, using a single-trait breeding goal constrained the reduction of eutrophication by 39.7% compared with a two-trait breeding goal weighted by ENV. Using a single-trait breeding goal could even increase eutrophication compared to the two-trait breeding goal weighted by ENV if the genetic correlation is higher than − 0.28.

Although %fat is acknowledged to be an important driver of the results obtained, we did not investigate the effect of a potential change of the correlation between %fat and TGC and FCR on selection response when the correlation between TGC and FCR changed. Mainly because we do not know precisely how the genetic correlations between three traits would behave when the genetic correlation between two of these traits would change. Nevertheless, if we consider that the genetic correlation between TGC and %fat is close to the 0.75 value tested here, the correlation between FCR and %fat would have a strong effect on the response to selection when the genetic correlation between TGC and FCR is weak. In that case, the response to selection for FCR would probably be higher if the genetic correlation between FCR and %fat is stronger.

So far, in fish, there are strong indications that the correlation between ${\text{TGC}}$ and ${\text{FCR}}$ is weak (between 0 and − 0.4, e.g. [26]). Hence, both traits (${\text{TGC}}$ and ${\text{FCR}}$) should be included in the breeding goal and in the index to maximize the economic or environmental responses. However, the success of a breeding program in improving ${\text{FCR}}$ largely depends on the availability of phenotypes that can be used as indirect criteria for ${\text{FCR}}$. To date there is no method to record ${\text{FCR}}$ efficiently at a low cost that have been implemented in a fish breeding program although several methods have been proposed [33, 34]. Therefore, finding an efficient method to phenotype fish for ${\text{FCR}}$ is an important challenge for fish breeders. In this regard, muscle fat content may be a trait of premium interest as it can be measured on selection candidates with non-invasive ultrasound measurements [35]. In the pig industry, Knap and Wang [36] reported positive genetic correlations between backfat depth and ${\text{FCR}}$, which means that selection for leaner pigs led to an improvement of ${\text{FCR}}$ because fat deposition is less efficient in terms of energy used per unit of wet weight gain than protein deposition. In fish, fat is mostly deposited as visceral and intramuscular/subcutaneous fat and it has been reported that fat content related traits and ${\text{FCR}}$ are genetically correlated [26, 37]. In 2007, Quillet et al. [38] showed that a trout line selected for low muscle lipid content was more efficient than a line selected for high muscle lipid content. In our study, we used muscle fat as an indirect criterion in the index based on results from Besson et al. [33]. Surprisingly, even an indirect criterion with a relatively weak genetic correlation with ${\text{FCR}}$ (− 0.39) resulted in a reduction in eutrophication. Thus, assuming that ${\text{TGC}}$ or another growth trait is always the main trait in the breeding goal, the inclusion in the index of any other indirect criterion with a strong correlation with ${\text{FCR}}$ would improve FCR and thus increase economic return and reduce eutrophication. However, other methods should be investigated such as weight loss after fasting [39] or individual FCR in aquarium under restricted feeding, which was shown to be phenotypically linked to ${\text{FCR}}$ [33].

We also explored what would be the best type of weighting factor for a two-trait breeding goal (with ${\text{TGC}}$ and ${\text{FCR}}$ in the breeding goal) to enhance the sustainability of fish production. We found that, when the correlation between ${\text{TGC}}$ and ${\text{FCR}}$ is strongly negative, the environmental response is not sensitive to the use of EV or ENV in the breeding goal. This is because the strong favourable genetic correlation between ${\text{TGC}}$ and ${\text{FCR}}$ brings information on the EBV of ${\text{FCR}}$ (the trait with the greatest relative EV and ENV), which enhances the favourable response of ${\text{FCR}}$. However, the response in economic return and in reduction of environmental impacts is sensitive to the use of EV versus ENV when the genetic correlation between ${\text{TGC}}$ and ${\text{FCR}}$ is weakly negative. First, although the reduction of eutrophication at the farm level is lower with EV than with ENV, it remains favourable because EV puts more emphasis on improving ${\text{FCR}}$ and results in a reduction of the amount of feed required per unit of fish produced. Thus, using EV maximizes the economic return but is also promising for reducing eutrophication. However, using ENV when the genetic correlation between TGC and FCR is weak decreases the economic return, i.e. by 56.4% compared to a breeding goal using EV when the correlation is − 0.2. The reason is that the ENV of ${\text{TGC}}$ and ${\text{FCR}}$ are both negative whereas the EV of ${\text{TGC}}$ is positive and the EV of ${\text{FCR}}$ is negative; this change causes a large shift in trait responses. With ENV, the main opportunity to reduce eutrophication is not to select for better ${\text{FCR}}$ but to reduce ${\text{TGC}}$. However, this makes no sense in economic terms because ${\text{TGC}}$ has a positive EV, and then the economic return of the breeding program decreases drastically. In this case, the financial incentive for farmers to decrease eutrophication by using ENV in the breeding goal is low and using ENV may not be the solution to enhance the sustainability of fish production. Thus, using ENV instead of EV depends on the willingness of farmers to accept a slightly lower increase in economic return in exchange of an improvement in environmental impacts. However, farmers could benefit from such an environmental-based breeding program indirectly, since it has been shown that consumers are willing to pay a price premium for salmon produced with more environmental considerations [40]. Thus, the potential increase in sale price could offset some of the lost economic return, as a result of using ENV. In practice, the local environmental impact of fish farming is also determined by spatial planning and can be managed by adapting the quota system.

If there is an antagonism between EV and ENV, it could be interesting to combine them in the aggregate genotype. However, this requires that they are expressed in the same units, i.e. that ENV is converted to a monetary unit. This is possible when ENV is calculated for climate change because a shadow price of carbon exists, which is defined as the cost of the damage caused by emitting an additional ton of CO_2_. Combining EV and ENV in the breeding goal would balance out the genetic gain between economic return and environmental impact [41, 42]. However, to our knowledge, other categories of impacts such as eutrophication have not yet been monetarized.

Our study shows that, although a quota is implemented to constrain the environmental impacts, environmental impacts per farm per year could increase as a result of genetic improvement, especially when only growth is improved. In that case, and assuming a weak correlation with ${\text{FCR}}$, improving ${\text{TGC}}$ would increase environmental impacts per farm per year, although the quota on biomass is respected. The aim of the quota on biomass is to ensure that the surrounding environment has the capacity to assimilate the nutrients produced by the farm, which is termed the carrying capacity of the environment [43]. Thus, although the emission of waste per day does not exceed the carrying capacity, it would be essential to verify that the local environment is not affected by the increase of the annual emission of wastes. In such a case, breeding would become a problem and not a solution to reduce environmental impacts of fish farming. To change this, the breeding program should be modified to respect the annual carrying capacity by including other traits in the breeding goal and in the index. For instance, in our situation, adding ${\text{FCR}}$ in the breeding goal and %fat in the index would reduce the amount of nutrients emitted per year per farm regardless of the weights used in the breeding goal. Another solution would be to change the overall quota regulation by imposing an annual quota on feed used. In such a case, it is likely that the EV and ENV of the traits would differ but ${\text{FCR}}$ would remain the key trait to be improved and this quota definition would motivate breeders to include it in their index. The importance of feed efficiency in breeding programs to reduce environmental impacts has also been demonstrated by Ali et al. [41, 42] in livestock. They showed that using EV that integrate environmental costs in a pig breeding program for growth and ${\text{FCR}}$ results in reducing greenhouse gas emissions and excretions of nitrogen and phosphorus.

## Conclusions

This is the first study that explores the influence of the genetic correlation between growth rate and feed conversion ratio on the optimal breeding program for economic or environmental sustainability. We showed that a favourable response in ${\text{FCR}}$ is key to improving profit and to reducing eutrophication at the farm level because it reduces the amount of feed used to produce one kg of fish. Feed is the largest economic cost for farmers and also the largest environmental cost due to its manufacturing and its biological transformation into nitrogen-based waste by the fish [44]. We showed that the two-trait breeding goal using with %fat in the index as indirect criterion of FCR was best to reach the favourable response in FCR. Using EV in this two-trait breeding goal increased economic return by 5 to 127% compared to a single-trait breeding goal for ${\text{TGC}}$. Furthermore, this two-trait breeding goal was able to reduce eutrophication by 1.34% (using ENV) and 0.63% (using EV) per kg of fish produced per year when the correlation between ${\text{TGC}}$ and ${\text{FCR}}$ is − 0.25. Based on these results, we strongly recommend to include ${\text{FCR}}$ in breeding goals with an indirect criterion in the index of a fish breeding program, especially if the correlation between ${\text{TGC}}$ and ${\text{FCR}}$ is weak.

## Supplementary information

**Additional file 1: Tables S1.** Calculations and parameters involved in the bio-economic model.

**Additional file 2: Tables S2.** Technical parameters of the sea bass farm running under a quota on biomass.

**Additional file 3: Tables S3.** Revenue and costs (variable and fixed) of a sea bass farm running under a quota on biomass.

## Data Availability

The R script used to compute the response to selection for several genetic correlation and the datasets generated for the current study are available from the corresponding author on request.
