# Supplementary material for: The genetic correlation between feed conversion ratio and growth rate affects the design of a breeding program for more sustainable fish production
Source: Genet Sel Evol. 2020 Feb 7;52:5. doi: 10.1186/s12711-020-0524-0 (PMC7006397; doi:10.1186/s12711-020-0524-0)
Supplement: Supplementary file 1 — Additional file 1: Tables S1. Calculations and parameters involved in the bio-economic model. [file 12711_2020_524_MOESM1_ESM.docx]

Additional Table S1: Calculations and parameters involved in the bio-economic model.

| Parameters | Formulas | |
| --- | --- | --- |
| Fish model |  | |
| Thermal growth coefficient (TGC) :  *1-b = weight exponent = 0.51*  *K_i_ = daily corrected temperature*  *W_H_ (harvest weight) = 13 g*  *W_I_ ( initial weight) = 1300 g*  *n is the length of growing period until harvest weight* | $TGC =\frac{W_{H}^{1-b} - W_{I}^{1-b}}{\sum_{i=1}^{n} K_{i}}$ | |
| Fish weight (W_n_) in kg : | ${W_{n}= [W_{I}^{0.51} + (TGC \times\sum_{i=1}^{n} K_{i})]}^{1/0.51}$ | |
| Daily weight gain (DWG_n_) in g : | $\mathrm{DWG}_{n} = W_{n} - W_{n-1}$ | |
| Feed conversion ratio (FCR_Wn_) in g/g : |  | |
| $\mathbf{FCR}_{\mathbf{Wn}}\boldsymbol{= \alpha\times}\frac{\mathbf{W}_{\mathbf{n}}^{\mathbf{0.14}}}{\mathbf{1.318-}\left( \boldsymbol{0.103\times}\mathbf{T}_{\mathbf{i}} \right)\mathbf{+}\left( \boldsymbol{0.007174\times}{\mathbf{T}_{\mathbf{i}}}^{\mathbf{2}} \right)\boldsymbol{-(0.0001395\times}{\mathbf{T}_{\mathbf{i}}}^{\mathbf{3}}\mathbf{)}}$ | | |
| Daily feed intake (DFI_n_) in g : | $\mathrm{DFI}_{n} = \mathrm{DWG}_{n} \times\mathrm{FCR}_{\mathrm{Wn}}$ | |
|  |  | |
| Daily dissolved N ($\boldsymbol{N\_dissolved}_{\mathbf{n}}$) in g: | ${N\_dissolved}_{n}= \mathrm{DWG}_{n}((65.988 \times\mathrm{FCR}_{\mathrm{Wn}})-25)$ | |
| Daily emission of P in effluent water (P_eff_n_) in g: | ${P\_eff}_{n}=00876\mathrm{DFI}_{n}-004 \mathrm{DWG}_{n}$ | |
|  |  | |
| Batch model |  | |
| Biomass of fish of 10 g stocked per batch (biomass_ini_)   - *Maximum_standing_stock = 435 tons* - *j = number of batch reared simultaneously (34)* - *biomass_i_ = biomass of batch i a maximum standing stock* | | ${biomass}_{ini}= \frac{maximum\_standing\_stock}{\sum_{i=1}^{j} {biomass}_{i}}$ |
|  |  | |
