# Supplementary material for: The genetic correlation between feed conversion ratio and growth rate affects the design of a breeding program for more sustainable fish production
Source: Genet Sel Evol. 2020 Feb 7;52:5. doi: 10.1186/s12711-020-0524-0 (PMC7006397; doi:10.1186/s12711-020-0524-0)
Supplement: Supplementary file 2 — Additional file 2: Tables S2. Technical parameters of the sea bass farm running under a quota on biomass. [file 12711_2020_524_MOESM2_ESM.docx]

Additional Table S2: Technical parameters of the sea bass farm running under a quota on biomass.

| **Parameters** | **Values** |
| --- | --- |
| Stocking weight | 0.01 kg |
| Harvest weight | 0.4 kg |
| Average morality over the production cycle | 10 % |
| Feed wasted | 10 % |
| Number of 600 m^3^ cages | 34 |
| Number of 1800 m^3^ cages | 34 |
| Density of transfer from 600 m^3^ cage to 1800 m^3^ cage | 10 kg/m^3^ |
| Quota on biomass | 435 tons |
