# Supplementary material for: The genetic correlation between feed conversion ratio and growth rate affects the design of a breeding program for more sustainable fish production
Source: Genet Sel Evol. 2020 Feb 7;52:5. doi: 10.1186/s12711-020-0524-0 (PMC7006397; doi:10.1186/s12711-020-0524-0)
Supplement: Supplementary file 3 — Additional file 3: Tables S3. Revenue and costs (variable and fixed) of a sea bass farm running under a quota on biomass. [file 12711_2020_524_MOESM3_ESM.docx]

Additional Table S3: Revenue and costs (variable and fixed) of a sea bass farm running under a quota on biomass.

| Item | Values |
| --- | --- |
| Variable revenue |  |
| fish | 5.60 €/kg of fish |
| Variable Costs |  |
| feed | 1.30 €/kg of feed |
| juveniles | 0.25 €/unit |
| Fixed costs |  |
| insurance | 185,000 €/farm/year |
| administration | 100,000 €/farm/year |
| labor | 1,500,000 €/farm/year |
| rent | 200,000 €/farm/year |
| maintenance | 60,000 €/farm/year |
| energy | 200,000 €/farm/year |
